# Function and Engineering of a Food Enzyme Under Coupled High-Temperature–Pressure Conditions: Insights from Molecular Dynamics Simulation and Experimental Validation

**DOI:** 10.3390/foods14142485

**Published:** 2025-07-16

**Authors:** Zidan Liu, Weihao Long, Keying Chen, Linyu Luo, Qiong Li, Tolbert Osire, Nan Zheng, Mengfei Long

**Affiliations:** 1 College of Pharmaceutical Sciences, Southwest University, Chongqing 400715, China; shmily0920@email.swu.edu.cn (Z.L.); long156836@email.swu.edu.cn (W.L.); cky210313@email.swu.edu.cn (K.C.); lly4566@email.swu.edu.cn (L.L.); liqiong8011@126.com (Q.L.); 2 Faculty of Biology, Shenzhen MSU-BIT University, 1 University Park Road, Shenzhen 518172, China; 6420210017@smbu.edu.cn; 3 Key Laboratory of Industrial Biotechnology of Ministry of Education, School of Biotechnology, Jiangnan University, Wuxi 214122, China

**Keywords:** ethyl carbamate hydrolase, molecular dynamics, temperature, pressure, enzyme function

## Abstract

The relationship between protein structure and function is intrinsically interconnected, as the structure of a protein directly determines its functional properties. To investigate the effects of temperature and pressure on protein function, this study employed ethyl carbamate (EC) hydrolase as a model food enzyme and conducted molecular dynamics (MD) simulations under varying temperature and pressure levels to elucidate its structure–function relationship. By systematically analyzing the dynamic changes in root mean square deviation (RMSD), root mean square fluctuation (RMSF), radius of gyration (Rg), solvent accessible surface area (SASA), hydrogen bonding, catalytic pocket conformation, and packing density under different temperature and pressure conditions, we revealed the structural adaptability of EC hydrolase. Furthermore, we analyzed the characteristics of EC hydrolase using molecular dynamics simulations with temperature and pressure levels, as well as conformational bias-based computer-aided engineering, providing both theoretical and experimental foundation for the adaptability mechanisms of enzymes under extreme conditions.

## 1. Introduction

The adaptation mechanism of enzymes to complex environments has been a “hotspot” area of active research [1]. The optimal temperature range for the activity and stability of enzymes in natural extremophiles could reach 80–100 °C, with these enzymes also exhibiting excellent stability under high hydrostatic pressures of 60 to 100 MPa [2]. Coincidentally, enzymes are often engaged in an extremely high-temperature and high-pressure environment for industrial application, which is hard to maintain stable during the processing, hindering their application. However, there seems to be no generally adapted mechanism, but a complicated solution, by combining several factors [3,4].

The conformation of enzymes may be significantly disturbed when temperature and pressure fluctuate [5,6]. The optimal temperature poses a balance between flexibility and rigidity, while increasing temperature will lead to unfolding or denaturation, causing loss of enzyme function [7,8]. Similarly, enzymes can also turn inactive when exposed to high pressure, due to collapse of internal cavities, resulting in unfolding [9]. In general, the binary relationship between flexibility and rigidity has been considered as a conceivable factor for stable enzymes [10,11,12,13]. Biochemical functions of some enzymes rely on their conformational flexibility, while the fluctuations of flexible residues in complex contexts lead to undesired structure folding. Therefore, rigidifying flexible residues may be an efficient approach to improve stability [14,15]. Besides the flexibility, oligomerization has also been reported to be critical to the stability of some enzymes [16,17]; however, the structure-based mechanisms behind this stability are still unclear.

The crystal structures of most stable proteins have been resolved at room temperature, rather than their natural temperature or pressure. Therefore, in order to fully understand the structure, dynamics and function of proteins, static structural determination needs be underpinned by various spectral techniques (e.g., small-angle X-ray scattering (SAXS) and nuclear magnetic resonance (NMR), as well as computer-aided simulations under different external conditions [18,19,20,21], which have become the most powerful methods to explore the energy landscape of proteins and their flexibility–function adaption relationships within accessibly natural conditions.

High-temperature molecular dynamics simulations have been widely used to sift through mutation pools to improve the activity and stability of enzymes. However, growing temperature would exacerbate molecular entropy and protein unfolding volume. Contrarily, the increase in pressure could lead to organized molecular order, with declining system entropy and decreased volume of protein unfolding at constant temperature [22]. Kitchen et al. reported one of the earliest molecular dynamics (MD) simulations of bovine trypsin inhibitors in water under variable pressure, ranging from 0.1 to 1000 MPa [23]. The result indicated that most of the protein structures were conservative at 1000 MPa, where the radius of gyration (Rg) and atomic fluctuations decreased, and the hydration of water molecules was more orderly. We presume that in-depth understanding of the effect of high temperature and high pressure on enzyme structure could not only gain insights into the adaptation mechanism of enzymes, but also be conductive to further guiding the modification of the enzyme.

In this study, we carried out a systematic temperature–pressure coupled MD simulations to investigate the effects of pressure and temperature on the structure and dynamic behavior in primary and non-native environments, taking ethyl carbamate (EC) hydrolase as an example; this a typical enzyme in industrial food applications to degrade EC, which is mainly found in fermented foods and fermented alcoholic beverages, causing carcinogenic potential to humans. EC hydrolase was selected for its globular structure, with sensitive internal regions ideal for studying conformational dynamics under varying temperature and pressure conditions. We soundly analyzed the changes in solvent-accessible surface area (SASA), Rg, interaction force, substrate binding pocket and packaging density of enzymes under different temperature–pressure conditions. We also provided the potential factors underlying the enzyme adaptability observed under high temperature and high pressure, which may provide further insight into adaptive evolution of enzymes in complex conditions such as toxic catalysts, organic solvents, high temperatures, or high pressures. Moreover, we integrated Conformational Biasing (CB) computational analysis to identify critical mutation sites under various temperatures and pressures, for experiment validation. This investigation elucidated the underlying factors contributing to EC hydrolase’s adaptability under high-temperature and high-pressure conditions, offering valuable insights into the adaptive evolution of enzymes in challenging contexts, including exposure to toxic catalysts, organic solvents, elevated temperatures, and high pressures, as detailed through subsequent computational and experimental analyses.

## 2. Materials and Methods

### 2.1. Molecular Dynamics Simulations at Coupled High-Temperature–Pressure

The MD simulations were carried out with the GROMACS 2023.4 (www.gromacs.org) [24]. A structure model for EC hydrolase was generated using AlphaFold3 [25]. During simulations, Amber99SB force fields and TIP4P water models were used. Enzymes were inserted into the box at least 1.0 nm from the box edge, and the box was defined as a cube. In order to neutralize the system, sodium ions were added. Energy minimization procedures based on steepest descent were applied to the systems [26]. The simulated temperature was set to 273 K, 288 K, 303 K, 318 K, and 333 K, and the pressure was set to 1 bar, 100 bar, 500 bar, 1000 bar, 2000 bar, and 4000 bar, respectively, with a time step of 2 fs. Each system was simulated for 60 ns, with three independent replicates.

### 2.2. MD Trajectory Analysis

Analyses including root mean square deviation (RMSD), root mean square fluctuations (RMSF), radius of gyration (Rg), solvent-accessible surface area (SASA), number of hydrogen bonds between protein and protein, and hydrogen bond changes between protein and water were calculated using GROMACS MD simulation packages. The reported structural metrics (RMSD, RMSF, Rg, SASA, etc.) were averaged over three independent MD simulation trajectories per system. Dynamic cross-correlation map (DCCM) analysis was performed using Bio3D. Protein packing density was calculated using an online server Voronoia 4-ever (https://proteinformatics.uni-leipzig.de/voronoia/ (accessed on 20 November 2024) [27].

### 2.3. Ananysis of Number of Water Molecules

The most representative conformation of each trajectory was selected, and then the number of water molecules was calculated using PyMoL 3.0.3. An analysis of the oxygen atom occupancies in the water molecules can determine the number of water molecules.

### 2.4. Substrate-Binding Pocket Analysis

The volume changes of the substrate-binding pocket were calculated, using MDpocket in fpocket2 software. With default parameters, fpocket2 was used to search the substrate-binding pocket of EC hydrolase [28]. The MD snapshots extracted from the trajectory were saved every 1 ps and aligned to a reference structure. Pocket volumes were calculated by MD pocket with –f flag and default settings.

### 2.5. Conformational Biasing Analysis

The Conformational Biasing method, implemented via a Colab-based pipeline (https://github.com/alicetinglab/ConformationalBiasing/tree/main (accessed on 10 May 2025)), combined with ProteinMPNN sequence optimization, enabled the design of EC hydrolase variants with tailored conformational state preferences. The process began by uploading custom PDB structures of the target protein, specifically stable clustered conformations derived from molecular dynamics (MD) simulations at 273 K/1 bar (State 1) and 333 K/4000 bar (State 2). Sequence alignment was performed using a PairwiseAligner to map residues across these structures, allowing optional sequence mismatches, and a consensus sequence was generated from the input PDB-derived sequences to identify residues present in both states for mutation analysis. ProteinMPNN processed PDB structures pre-treated with PyMOL (removing non-protein atoms and adding hydrogens), using the wild-type sequence as a baseline, and ran with a fixed backbone design and default parameters to generate 100 mutant sequences per structure. The algorithm calculated log-probability scores for each sequence using a neural network model, converting logits to a probability distribution via the softmax function to reflect sequence stability in specific conformations. Mutant sequence scores were computed relative to the wild-type score (Δscore) and normalized to produce standardized scores, which were used to compare mutant biases toward State 1 or State 2, identifying the top 5% of mutants as conformationally biased or neutral. Results were visualized in a scatter plot to highlight mutants with significant conformational bias for further analysis.

### 2.6. Strains, Plasmids, and Culture Conditions

The EC hydrolase gene from *Lysinibacillus fusiformis* SCO2 (GenBank ID: KU353448), originally identified in the murine gastrointestinal tract, was synthesized by Sangon Biotech and subsequently cloned into the pET-20b vector for expression in *Escherichia coli* BL21(DE3). Site-directed mutagenesis of the wild-type plasmid was conducted using the Fast Mutagenesis Kit V2, with the original EC hydrolase plasmid serving as the template. The primer sequences employed are detailed in Table 1. Both Luria–Bertani (LB) and Terrific Broth (TB) media were employed for cultivation and protein expression. Recombinant strains were initially cultured in 20 mL of LB medium supplemented with 100 μg/mL ampicillin at 37 °C overnight. Subsequently, 3% (*v*/*v*) of the seed culture was inoculated into 200 mL of TB medium and incubated at 37 °C. Upon reaching an optical density at 600 nm (OD_600_) of 0.4, isopropyl β-D-1-thiogalactopyranoside (IPTG) was added to a final concentration of 0.1 mmol/L, followed by induction for 18 h at 25 °C.

### 2.7. Enzyme Purification

Following protein expression, bacterial cells were collected via centrifugation and subsequently resuspended in a phosphate buffer. Cell lysis was achieved through sonication at 4 °C for 30 min, after which centrifugation was employed to isolate the supernatant. His-tagged proteins were subsequently purified using nickel affinity chromatography. During this purification process, non-specifically bound proteins were removed with 100 mM imidazole, whereas the target protein was eluted with approximately 300 mM imidazole. The fractions containing the target protein were concentrated and desalted using ultrafiltration tubes. The resulting protein solution was then appropriately diluted and utilized for subsequent enzymatic activity assays.

### 2.8. Determination of Enzymatic Activity

The enzymatic activity of EC hydrolase was evaluated by measuring the ammonia released during the reaction. One unit of enzymatic activity was defined as the quantity of enzyme required to catalyze the formation of 1 μmol of NH_3_ per minute under optimal assay conditions. For the assay, 1 mL of enzyme solution was mixed with 1 mL of a 3% (*w*/*v*) ethyl carbamate (EC) solution and incubated at 37 °C for 15 min. The reaction was terminated by the addition of 1 mL of 10% trichloroacetic acid. Subsequently, 1 mL of Solution I (comprising 15 g of phenol and 0.625 g of sodium nitroprusside dissolved in 250 mL) and 1 mL of Solution II (containing 13.125 g of sodium hydroxide and 7.5 mL of sodium hypochlorite in 250 mL) were added. The mixture was then incubated at 37 °C for an additional 20 min. The absorbance at 625 nm was measured to determine the ammonia concentration.

The enzyme activity was calculated as

(1) $$\mathrm{enzyme}\;\mathrm{activity}=∆{OD}_{625}\times n\times \frac{k}{15}$$

where ΔOD_625_ represents the absorbance difference between the sample and the blank, n denotes the dilution factor of the enzyme solution, k is the reciprocal of the slope of the standard curve, and 15 is the reaction time in minutes.

### 2.9. Ethanol-Tolerance Assay

The analysis of ethanol tolerance involved assessing the impact of varying ethanol concentrations (ranging from 0 to 25% *v*/*v*) on protein activity. Protease activity was measured following a 1 h incubation period at these ethanol concentrations, to evaluate the enzyme’s stability in the presence of ethanol.

### 2.10. Evaluation of Enzymatic Performance in a Simulated Wine System

To evaluate the EC-degrading efficacy of both wild-type and mutant enzymes under conditions closely resembling real-world scenarios, experiments were conducted using a model wine solution. This solution comprised 500 μg/L of ethyl carbamate, 15% (*v*/*v*) ethanol, and was adjusted to a pH of 4.5. Protein samples were introduced into the synthetic wine matrix, representative of yellow wine, at a final concentration of approximately 1.5 mg/mL. The reaction mixture was incubated at 30 °C for a duration of 12 h. Samples were collected at intervals of 1, 3, 7, and 12 h to determine the residual EC content. The concentration of ethyl carbamate in each sample was quantified using gas chromatography–mass spectrometry (GC-MS; Thermo Fisher Scientific, Austin, TX, USA).

## 3. Results and Discussion

### 3.1. Molecular Dynamics Simulations of Hydrolase at Different Temperature and Pressure

**Figure 1:**
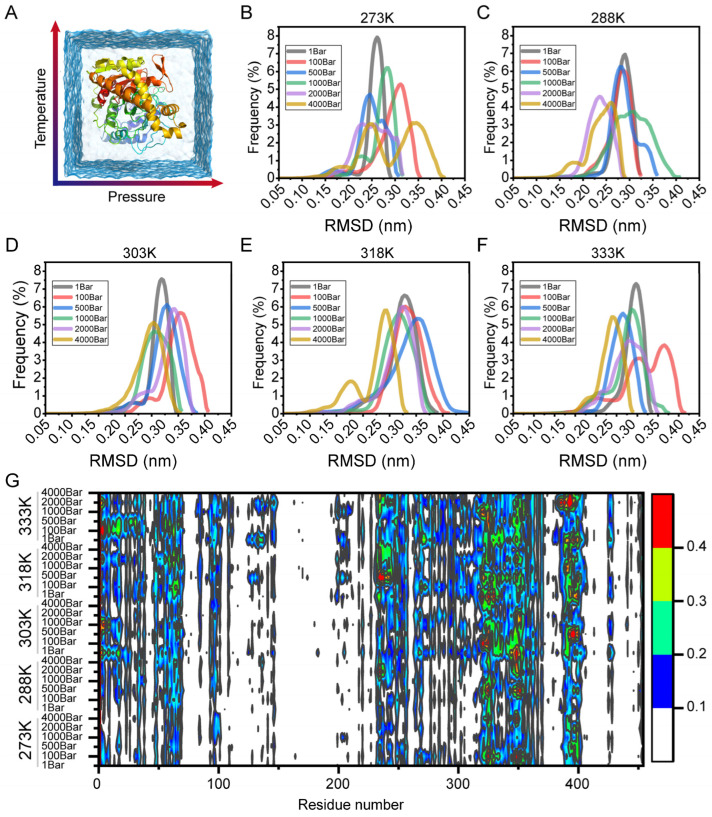
(**A**) Schematic illustration of MD simulations under different temperatures and pressures. (**B**–**F**) RMSD values of EC hydrolase at 273 K, 288 K, 303 K, 318 K, and 333 K under different pressures (1 bar, 100 bar, 500 bar, 1000 bar, 2000 bar, 4000 bar). (**G**) RMSF values of EC hydrolase at 273 K, 288 K, 303 K, 318 K, and 333 K under different pressures (1 bar, 100 bar, 500 bar, 1000 bar, 2000 bar, 4000 bar).

The structural flexibility of proteins is associated with numerous biological functions, including catalytic activity, substrate binding, and molecular recognition. To investigate the flexibility, we conducted molecular dynamics simulations at five different temperature levels and six pressure levels, each lasting for 60 ns (Figure 1A). The overall flexibility of the protein structure was assessed by calculating the backbone RMSD for all residues (Figure 1B–F). The overall RMSD distribution showed differences in the number of sampled conformational states under each simulation condition. Notably, high-temperature and high-pressure conditions exhibited a significantly higher number of distinct conformational states compared to WT. Furthermore, we observed a minor population of a second conformational state in the RMSD distribution under non-normal conditions (Figure 1B). The increased number of conformational states observed in simulations may indicate selective local unfolding or stabilization within specific structural elements, which are distinct from global structure changes. Consequently, we analyzed the RMSF values across all states to identify regions most responsive to temperature and pressure.

To gain insights into which structural features remain stable or become unstable in comparison to the initial structural dynamics and stability of their respective parent structures, we investigated the normalized RMSF values of the protein under all conditions (Figure 1G). The heat map indicated that these variants had little impact on the flexibility of the majority of structures. Conversely, the effects of temperature and pressure increased (red in Figure 1G) or decreased (blue in Figure 1G) the flexibility of a limited number of local structural regions. This demonstrated a potential temperature and pressure pathway through which regions outside the active site, with their dynamic correlation, can provide a favorable target for stable mutations, even if the target sites are not identified as the most flexible regions.

### 3.2. Tightness Changes at Different Temperatures and Pressures

**Figure 2:**
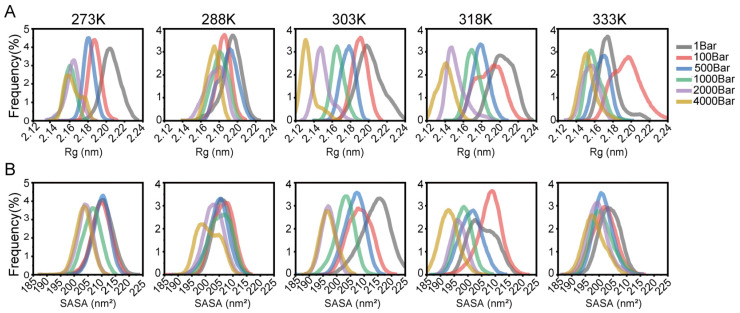
(**A**) Rg values of EC hydrolase at 273 K, 288 K, 303 K, 318 K, and 333 K under different pressures (1 bar, 100 bar, 500 bar, 1000 bar, 2000 bar, 4000 bar). (**B**) SASA values of EC hydrolase at 273 K, 288 K, 303 K, 318 K, and 333 K under different pressures (1 bar, 100 bar, 500 bar, 1000 bar, 2000 bar, 4000 bar).

The Rg describes the distance between the axis and the maximum inertia point in the rotating system, representing a crucial physical parameter that characterizes the tightness of the protein structure. A smaller radius indicated a more tightly packed conformation. A slight increase in the radius suggested that conformational extension is due to the movement of the unfolded part of the enzyme. As depicted in Figure 2A, at lower temperatures, the distribution of Rg closely followed the trend of increasing pressure, although there is not a clearly defined limit, particularly noticeable at 288 K. As the temperature gradually approached the optimal temperature, the influence of pressure on Rg became most prominent. At 303 K and 318 K, the Rg of enzymes under different pressures displayed a noticeable gradient. However, the influence of pressure on Rg decreased at the higher temperature of 333 K. Therefore, we propose that the pressure sensitivity of urethane hydrolase is strongest near the optimal temperature.

To further investigate the effects of varying temperatures and pressures on protein surface characteristics, we also assessed the SASA. SASA is defined as the surface area traced by the center of a solvent molecule with a radius of 0.14 nm as it rolls over the van der Waals surface of a protein. Hydrophobic groups tend to be buried in the protein far away from the solvent during the folding process, so the distribution of water molecules on the protein surface can be obtained by calculating the hydrophobic and hydrophilic SASA of the protein. An increase in the hydrophilic accessible surface area over the course of the simulation indicated an expansion of the hydrophilic cavity area on the protein’s surface and an increase in the number of water molecules within the cavity. Otherwise, a decrease illustrated that the protein has undergone a collapse, expelling water molecules from the cavity, which is inversely proportional to the extent of natural contact. The results of SASA analysis were basically consistent with the trend of Rg (Figure 2B). SASA showed no obvious relationship with different pressures at 273 K, 288 K, and 333 K. However, at temperatures around 303 K and 318 K, the SASA values for enzymes exhibited a distinct gradient and positive correlation with pressure.

### 3.3. Hydrogen Bond Changes at Different Temperatures and Pressures

**Figure 3:**
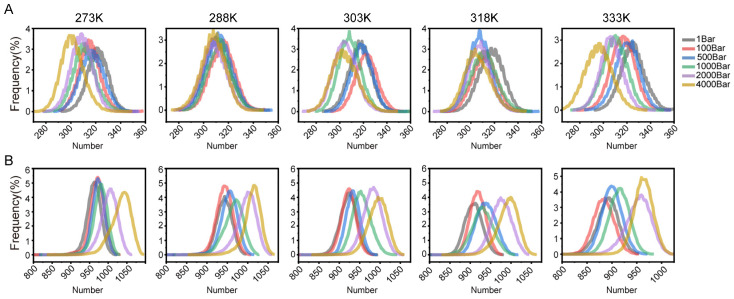
(**A**) Number of hydrogen bonds between EC hydrolase and water molecules. (**B**) Number of intramolecular hydrogen bonds within EC hydrolase.

To further quantify protein compactness, we calculated the number of hydrogen bonds (HBs) within the EC hydrolase and water molecules. As can be seen from the Figure 3A,B, it was observed that the number of HBs between residues decreased with increasing pressure, regardless of the temperature, possibly due to the intrusion of solvent affecting the formation of HBs. Moreover, around the optimal temperature of 303 K, a clear watershed was formed between low and high pressures. For the enzyme and water, it was found that, regardless of the temperature, the number of hydrogen bonds formed between the enzyme and the solvent decreased with higher pressure, with a more significant decrease observed at 2000 and 4000 bar. This phenomenon may be attributed to the increased compactness of the enzyme structure under high pressure. The augmentation of pressure is correlated with a diminution in the intensity of electrostatic or Coulomb interactions [29]. The hydration of water molecules on charged groups was intensified under pressurized conditions but attenuated at elevated temperatures. This phenomenon was substantiated by molecular dynamics simulations of the bovine pancreatic trypsin inhibitor (BPTI) conducted by Kitchen et al., which revealed substantial alterations in the structural hydration shell under high pressure [23]. The hydration shell was observed to be more ordered at elevated pressures, with high pressure inducing the maximum alignment of nonpolar surface groups. The catalytic activity of enzymes in organic media was contingent upon the hydration state of the enzyme, a state that was substantially influenced by pressure. Mozhaev et al. posited that, during the initial phase of thermal inactivation, proteins may lose some essential water molecules, a deprivation that could precipitate a structural reconfiguration [30]. The application of high hydrostatic pressure (HHP) may impede this process by enhancing the hydration of both charged and nonpolar groups. In essence, the application of high hydrostatic pressure strengthens the hydration shell of the protein. The study by Gabriel et al. revealed that the presence of acetone in the reaction medium resulted in a distinct lag phase in amyloid formation. This finding suggests that acetone creates an unfavorable microenvironment for amyloid fibril formation, impeding the organization of denatured proteins into fibrous structures [31,32].

### 3.4. The Flexibility of Catalytic Pockets and Their Interaction with Water

**Figure 4:**
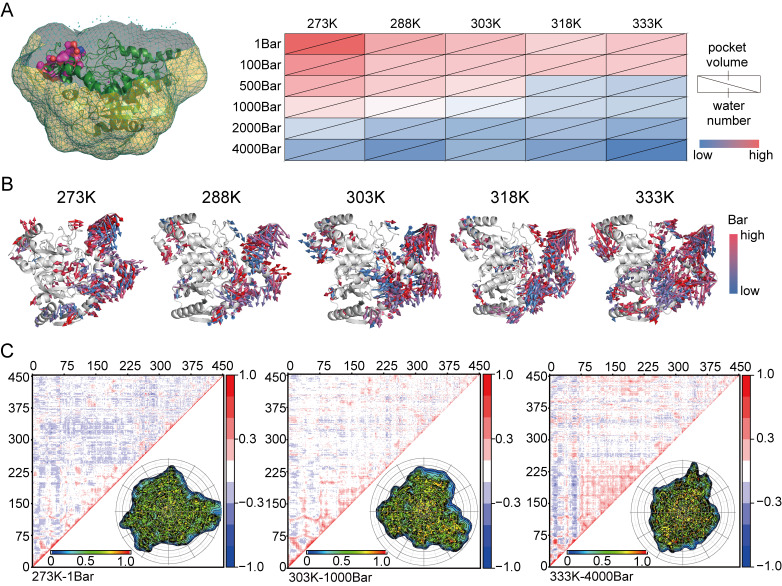
(**A**) The pocket volume, number of hydrogen bonds between EC hydrolase and water molecules and intramolecular hydrogen bonds within EC hydrolase. (**B**) Conformational changes in EC hydrolase under different temperatures and pressures. (**C**) Changes in packing density of EC hydrolase under different temperatures and pressures.

The protein interior cavity is considered to be crucial for the stability and function of proteins, and it has been reported that water molecules buried within these cavities affect the temperature- and pressure-mediated unfolding [33,34,35,36]. We calculated the volume of the completely buried cavities within the protein and the number of water molecules enclosed (Figure 4A). At lower temperatures, the substrate-binding pocket decreased in an orderly manner with increasing pressure; however, at 303 K and 318 K, a noticeable rebound in the substrate-binding pocket was observed at higher pressures of 1000 bar, known as the pressure activation effect. This phenomenon was consistent with the trend in the number of molecules in the substrate pocket. However, for enzymatic reaction systems under high pressure, the enzyme molecules exist in multiple equilibrium states: for instance, the balance between subunit dissociation and association; the balance between substrate binding and product release; and the ionization balance, and hydration balance between enzyme molecules. Therefore, the equilibrium constants measured under high pressure were not determined by a single factor, but were collectively determined by a series of factors. The partial molar volume of proteins dissolved in water was composed of the van der Waals volume of all atoms in the protein, the volume of cavities presented in the protein, and the volume of the hydration layer. Under normal circumstances, the van der Waals volume of all atoms can be neglected during pressure treatment, while the volume of cavities will decrease under pressure, and the volume of the hydration layer will increase under pressure. Finally, we analyzed the packaging density of ethyl carbamate hydrolase under varying temperatures and pressures, as shown in Figure 4B,C. The average packaging density generally displayed an increasing trend.

### 3.5. Engineering and Functional Characterization of EC Hydrolase Mutants with Conformational Biasing

**Figure 5:**
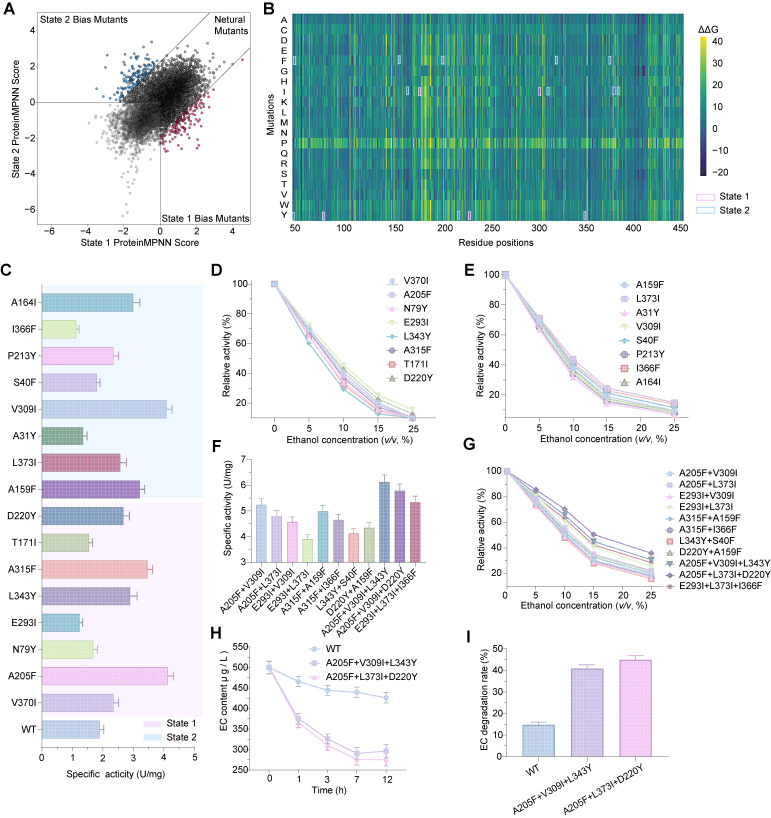
(**A**) Classification of mutants based on Conformational Biasing computational analysis, utilizing molecular dynamics simulations to delineate two conformational states—State 1 (normal conditions) and State 2 (high-temperature, high-pressure conditions), with ProteinMPNN scoring identifying key mutation sites. (**B**) Heatmap from Pythia-based virtual saturated mutagenesis, spanning residue positions 50 to 450, with free energy changes (ΔG) from −20 to +20 kcal/mol, where pink and blue regions indicate reduced ΔG for State 1 and State 2, respectively. (**C**) Specific activities for single-point mutants (pink regions indicated mutants of State 1 and blue region represented mutants of State 2). (**D**,**E**) Ethanol tolerance curves for selected mutants from State 1 and State 2, respectively. (**F**) Specific activities for double and triple mutants. (**G**) Ethanol tolerance for double and triple mutants. (**H**) EC degradation over 12 h. (**I**) EC degradation rates at 12 h. Error bars represent standard errors from triplicate experiments.

The classification of EC hydrolase mutants relied on computational analysis employing Conformational Biasing (CB) [37], a method that integrated molecular dynamics simulations to assess the enzyme’s structure under normal and high-temperature, high-pressure conditions, delineating two distinct states (State 1 and State 2) (Supplementary Material S1). ProteinMPNN scoring revealed a distribution of predicted mutants, with high-scoring variants clustering within a range of −6 to +4, indicating their potential significance for structural stability and functionality (Figure 5A). Low-scoring mutants, with scores below −2, dispersed widely, suggesting possible detrimental effects on enzyme performance. Moreover, we conduced virtual saturated mutagenesis to generate a heatmap spanning residual changes in free energy (ΔG), ranging from −20 to +20 kcal/mol, where pink regions indicated reduced ΔG and enhanced stability for State 1, and blue regions signifued reduced ΔG for State 2 (Figure 5B). These mutant candidates were chosen for subsequent experimental validation. The specific activities of single-point mutants from State 1 and State 2, as shown in Figure 5C, demonstrated notable improvements over the wild-type (WT) enzyme, which exhibited a specific activity of 1.89 U/mg. Mutants such as A205F achieved 4.12 U/mg, representing a 2.18-fold increase, while V309I reached 4.09 U/mg, a 2.16-fold enhancement. Other variants, including L373I at 2.57 U/mg, also showed moderate gains. For ethanol tolerance across 0–25% (*v*/*v*) ethanol concentrations, the WT enzyme retained 8.20% relative activity at 20% ethanol. In contrast, State 1 mutant E293I maintained 15.79%, a 1.93-fold improvement, and State 2 mutant L373I achieved 14.90%, a 1.82-fold increase (Figure 5D,E). Additional mutants, such as A205F (9.57%) and V309I (8.12%), exhibited moderate tolerance, showing a less pronounced decline than WT, validating the efficacy of the predicted mutation sites and providing a basis for further optimization.

The specific activities of double- and triple-mutant combinations, as shown in Figure 5E, underscored significant enhancements compared to single-point mutants and WT. Double mutants, such as A205F+V309I, attained 5.23 U/mg, a 27% increase over the best single mutant, while A205F+L373I reached 4.79 U/mg, a 16% improvement. Triple mutants further elevated performance, with A205F+V309I+L343Y achieving 6.12 U/mg, a 17% gain over A205F+V309I, A205F+L373I+D220Y at 5.79 U/mg (SE 0.256), a 21% rise, and E293I+L373I+I366F at 5.35 U/mg, a 37% enhancement. All combinations surpassed WT, with standard errors ranging from 0.18 to 0.28, highlighting the synergistic effects of multiple-site mutations and the superior catalytic potential of triple mutants. Ethanol tolerance profiles for double and triple mutants also demonstrated marked improvements (Figure 5G). The double mutant A205F+L373I retained 22.35% relative activity at 20% ethanol, a 2.73-fold increase over WT (8.20%), while E293I+L373I achieved 25.68%, a 3.13-fold enhancement. Triple mutants exhibited even greater resilience, with A205F+V309I+L343Y maintaining 30.68% at 20% ethanol, a 3.74-fold improvement, A205F+L373I+D220Y reaching 35.90%, a 4.38-fold gain, and E293I+L373I+I366F sustaining 28.46%, a 3.47-fold increase.

The WT enzyme reduced EC from 500 μg/L to 426.17 μg/L, yielding a degradation rate of approximately 14.77% (Figure 5H). The reference mutant H68A/K70R/S325N decreased to 337.81 μg/L, achieving a 32.44% degradation rate. Triple mutants A205F+V309I+L343Y and A205F+L373I+D220Y exhibited superior performance, declining to 295.90 μg/L and 275.46 μg/L, respectively, corresponding to degradation rates of 40.82% and 44.91%. The curves indicated rapid initial degradation within 1–3 h (from 500 to 310–325 μg/L), followed by a gradual decline from 7–12 h, with standard errors ranging from 11.50 to 16.50, aligning with reference data and reflecting enhanced degradation efficiency. The 12 h EC degradation rates further quantified these improvements (Figure 5I). The WT rate stood at 14.77%, while H68A/K70R/S325N reached 32.44%. The triple mutant A205F+V309I+L343Y achieved 40.82%, a 2.77-fold increase over WT, and A205F+L373I+D220Y attained 44.91%, a 3.04-fold enhancement. The consistent standard errors underscored the reproducibility of these results, highlighting the substantial potential of these mutants for practical EC degradation, particularly in industrial settings with high ethanol concentrations.

## 4. Discussion

In this study, we compared the structural and dynamic properties of urethane hydrolase using MD simulations that simulated conditions representative of both deep ocean and environmental settings. The pressure levels were selected to systematically investigate both biological and extreme conditions: (i) the 1–1000 bar range covers physiological pressures (1 bar standard, 100–500 bar deep-sea environments) and known protein transition zones; (ii) the 1000–4000 bar range probes forced denaturation thresholds while avoiding force-field limitations (>5000 bar). The results revealed that, as temperature and pressure increase to a certain extent, the enzyme adopted a more compact structure, accompanied by a decrease in the number of hydrogen bonds within the catalytic pocket. Furthermore, the state of pressure activation became evident at the optimal temperature and under relatively high-pressure conditions.

It is sometimes assumed that the overall structural flexibility of a protein is related to its activity, and RMSD is commonly employed to quantify this [37]. Based on the assumption that enhanced rigidity is the foundation for increased thermal stability, a general framework for understanding the stability and function of hyperthermophilic proteins under their natural conditions has been proposed. Other experimental and computational studies have reported that hyperthermophilic proteins exhibited greater conformational flexibility compared to their thermophilic congeners; however, varying pressures do not always present similar results [38,39].

In addition, a decrease in the number of water molecules within the enzyme’s buried cavity was observed under all conditions. It has been reported that the reduction in the volume of buried cavities was an adaptation mechanism under high-temperature conditions (thermal stability) since large solvent-filled cavities were known to lead to protein denaturation under high-temperature and high-pressure conditions [40]. In our previous studies, we developed a methodology involving MD simulations under six pressure levels (1 bar, 100 bar, 500 bar, 1000 bar, 2000 bar, 4000 bar) and selected four representative conformations from the simulated trajectory for cavity identification and calculation. The key amino acids that constitute the cavity were selected for virtual saturation mutation, and free energy calculations were performed by FoldX suite 5.0 and Rosetta 3.10 to screen five potentially effective mutants [35]. This study revealed that high pressure was used to select the target amino acids that constitute the cavity, and free-energy screening criteria were used to select suitable mutants, which provided a feasible strategy for improving the stability of proteins.

High temperature, chemical agents, and high pressure can all induce protein denaturation. While high temperature and chemical agents typically cause irreversible protein unfolding through covalent bond cleavage or molecular aggregation, high pressure primarily affects the tertiary and quaternary structures of proteins, with negligible impact on covalent bonds. Pressure modulates hydrogen bonds, electrostatic interactions, and hydrophobic interactions between amino acids and between amino acids and the solvent, driving EC hydrolase toward a more compact conformation, thereby influencing catalytic reaction rates. Changes in reaction rates can be attributed to: (i) direct alterations in enzyme structure; (ii) changes in the reaction mechanism, such as redefinition of the rate-limiting step; and (iii) changes in the physical properties of substrates or solvents (e.g., pH, density, viscosity) that affect enzyme structure or the rate-limiting step. In enzyme-catalyzed reactions, the reaction rate is governed by the slowest rate-limiting step, and ultra-high pressure (1–4000 bar) can redefine this step by inducing conformational changes, resulting in a nonlinear relationship between reaction rate and pressure. Consequently, MD simulations under varying pressure (1–4000 bar) and temperature conditions enable the capture of dynamic conformational changes in urethane hydrolase under diverse environmental conditions, providing a molecular basis for identifying sites that enhance stability or activity.

Through Conformational Biasing analysis, we further compared conformational states under different temperature and pressure conditions. Under high-pressure and high-temperature conditions, the enzyme favors a compact conformation, characterized by reduced hydrogen bond numbers, decreased RMSF, and diminished buried cavity volume in the catalytic pocket. This compact conformation enhances thermal stability by reducing solvent-filled cavity volumes, thereby mitigating denaturation. Compared to conformational ensemble analyses under ambient pressure, high-pressure and high-temperature MD simulations uniquely reveal transient compact conformations and pressure-induced solvent interaction changes. Additionally, Conformational Biasing analysis facilitates the identification of sites that enhance stability or activity by amplifying the sampling of favorable conformational states.

The integration of Conformational Biasing further elucidated the role of conformational states in modulating enzyme function, with the identification of high-scoring mutants with ProteinMPNN scores ranging from −6 to +4 across State 1 and State 2. Experimental validation confirmed that single-point mutants such as A205F and V309I achieved specific activities of 4.12 U/mg and 4.09 U/mg—respectively, 2.18- and 2.16-fold higher than the wild-type (WT) value of 1.89 U/mg—while exhibiting enhanced ethanol tolerance. The subsequent development of double and triple mutants, with A205F+V309I+L343Y reaching 6.12 U/mg and 30.68% ethanol tolerance, and A205F+L373I+D220Y achieving 5.79 U/mg and 35.90% tolerance, demonstrated synergistic enhancements, up to 3.04-fold in EC degradation efficiency (44.91% at 12 h). These findings extend beyond mere stability improvements, offering a strategic framework for enzyme engineering in complex industrial environments. The observed correlation between structural compactness and functional stability under high pressure suggests that rigidifying flexible residues, as seen in the reduced RMSF and increased packing density, could mitigate unfolding, a hypothesis supported by prior studies on hyperthermophilic proteins. However, the pressure activation effect and the role of hydration dynamics indicate that stability is not solely rigidity-dependent, but involves a delicate balance of conformational flexibility and solvent interactions. The superior EC degradation rates (40.82% and 44.91% for triple mutants versus 14.77% for WT) in a simulated wine system with 15% ethanol underscore the practical applicability of these mutants, particularly in mitigating ethyl carbamate accumulation in alcoholic beverages. Nevertheless, challenges remain, including the need to elucidate the precise molecular interactions driving the pressure activation effect and to optimize mutation combinations for broader environmental tolerances. Future research should explore longer simulation timescales and additional spectroscopic techniques (e.g., NMR, SAXS) to refine these insights, potentially integrating machine learning to predict multi-site interactions more comprehensively. This study thus establishes a robust foundation for leveraging computational and experimental synergies to engineer enzymes with enhanced stability and catalytic efficiency, paving the way for their industrial-scale deployment in challenging conditions.

## 5. Conclusions

This study conducted a comprehensive investigation into the structural and functional adaptations of ethyl carbamate hydrolase under varying temperature and pressure conditions through systematic molecular dynamics simulations, complemented by computational predictions and experimental validations. The research elucidated the effects of temperature and pressure on its conformational dynamics, solvent interactions, and catalytic pocket stability across native and non-native environments. The integration of computational design successfully identified key mutation sites, with experimental results demonstrating significant enhancements in specific activity (up to 6.12 U/mg for triple mutant A205F+V309I+L343Y), ethanol tolerance (up to 35.90% at 20% ethanol for A205F+L373I+D220Y), and EC degradation efficiency (up to 44.91% at 12 h). These findings provided a robust theoretical and empirical framework for the rational engineering of enzyme stability and performance, offering valuable insights for optimizing adaptability in complex food industrial settings such as high-temperature, high-pressure, and organic environments.

## Figures and Tables

**Table 1:** Primers for mutants.

Name	Primers
V370I-F	5′-TACAGAGATTTTTAATAAGATTGATGTGCTCATTTCCCCTAC-3′
V370I-R	5′-GGGTAGGGGAAATGAGCACATCAATCTTATTAAAAATCTCTG-3′
A205F-F	5′-CGGCTGCTACCCGCTATTTTGGAGCTTAGACCATATTGGTCC-3′
A205F-R	5′-TCGGACCAATATGGTCTAAGCTCCAAAATAGCGGGTAGCAGC-3′
N79Y-F	5′-CATGGGATACCTATGGCCTTAAAGGACTACTTGTATTTTAAA-3′
N79Y-R	5′-ATCTTTAAAATACAAGTAGTCCTTTAAGGCCATAGGTATCCC-3′
E293I-F	5′-GGATTCAGGCGCTAAAGTAATTGTGGTGCGTATTCCTTCCCT-3′
E293I-R	5′-GGGAAGGAATACGCACCACAATTACTTTAGCGCCTGAATCCA-3′
L343Y-F	5′-GAGCTTGGTGAGTACCCTTCTGCAGTCGATTACTTGCAGGCT-3′
L343Y-R	5′-AGCCTGCAAGTAATCGACTGCAGAAGGGTACTCACCAAGCTC-3′
A315F-F	5′-GGGCAGAGCTTGTGACGTCTCTTTCAGAGTTTGCAGCTATAC-3′
A315F-R	5′-GGTGTATAGCTGCAAACTCTGAAAGAGACGTCACAAGCTCTG-3′
T171I-F	5′-GGAGCAAGTGTTGCTTCACTAGGGATTGATACAGCAGGCTCT-3′
T171I-R	5′-GCCTGCTGTATCAATCCCTAGTGAAGCAACACTTGCTCCAGC-3′
D220Y-F	5′-GGTCCGATGACAAAGACAGTTAAGTACGCAGCGGGCTTGCTC-3′
D220Y-R	5′-GCAAGCCCGCTGCGTACTTAACTGTCTTTGTCATCGGACCAA-3′
A159F-F	5′-GTGGAGGCTCTGGTGCGTTTGTTGCAGCTGGAGCAAGTGTTG-3′
A159F-R	5′-GCAACACTTGCTCCAGCTGCAACAAACGCACCAGAGCCTCCA-3′
L373I-F	5′-GGTAGATGTGATAATTTCCCCTACCCTACCTATTGTAGCTAG-3′
L373I-R	5′-GCTACAATAGGTAGGGTAGGGGAAATTATCACATCTACCTTA-3′
A31Y-F	5′-CCCGTGGAATTAACGAAAGCTATTTTAGATTTTTACGAGGAA-3′
A31Y-R	5′-AGCCTGCAAGTAATCGACTGCAGAAGGGTACTCACCAAGCTC-3′
V309I-F	5′-GCAGAATGGGCAGAGCTTATTACGTCTCTTTCAGAGGCAGCA-3′
V309I-R	5′-GCTGCTGCCTCTGAAAGAGACGTAATAAGCTCTGCCCATTCT-3′
S40F-F	5′-CCTAAAATTAATTTTTATATGGCTTTTTATCGGGAAGAAGCC-3′
S40F-R	5′-GCTAAGGCTTCTTCCCGATAAAAAGCCATATAAAAATTAATT-3′
P213Y-F	5′-CCCGCTAGCATGGAGCTTAGACCATATTGGTTACATGACAAA-3′
P213Y-R	5′-TCTTTGTCATGTAACCAATATGGTCTAAGCTCCATGCTAGCG-3′
I366F-F	5′-CAGAGTTTTTTAATAAGGTAGATGTGCTCATTTCCCCTACCC-3′
I366F-R	5′-GGGGAAATGAGCACATCTACCTTATTAAAAAACTCTGTAAAC-3′
A164I-F	5′-4GGCTCTGGTGCGGCTGTTGCAGCTGGAATTAGTGTTGCTTCA-3′
A164I-R	5′-TAGTGAAGCAACACTAATTCCAGCTGCAACAGCCGCACCAGA-3′

## Data Availability

The original contributions presented in the study are included in the article/Supplementary Materials; further inquiries can be directed to the corresponding authors.

## Supplementary Materials

The following supporting information can be downloaded at: https://www.mdpi.com/article/10.3390/foods14142485/s1. Supplementary Material S1. ProteinMPNN scores of single mutants for Conformational Bias analysis.
